# An analysis model of torque dependence of critical current in a Bi2223/Ag composite concentric cylinder

**DOI:** 10.1186/s40064-016-3168-3

**Published:** 2016-09-08

**Authors:** Z. Yan, J. Jin, W. J. Feng

**Affiliations:** 1 Department of Engineering Mechanics, Shijiazhuang Tiedao University, Shijiazhuang, 050043 People’s Republic of China; 2 School of Civil Engineering, Hebei University of Science and Technology, Shijiazhuang, 050018 People’s Republic of China

**Keywords:** Degradation behavior of critical current, Linear weakening model, Elastic–plastic deformation, Torque dependence, Bi2223 composite superconducting cylinder

## Abstract

In this paper, a model of torque dependence of critical current in a Bi2223/Ag composite concentric cylinder is analyzed. Based on the hypothesis of planar section and elastic linear strengthening constitutive theory, the relation between torque and shear strain is firstly obtained. Then a linear weakening relation between critical current density and shear strain are put forward. Finally the effects of the applied torque together with the mechanical properties of superconducting core on the degradation behavior of critical current are calculated and discussed. The results and conclusions should be helpful to the application of superconducting materials subjected to elastic–plastic torsional deformation.

## Background

Critical current is an important characteristic of superconducting materials, and it is greatly influenced by mechanical deformation. Up to date, numerous theoretical and experimental studies on critical current in Bi2223 composite tapes with bending and/or tension strains have been made (Osamura et al. 2003; Katagiri et al. 2003; Sugano and Osamura 2004; Kuroda et al. 2005; Ochiai et al. 2008, 2010; Gou and Shen 2012; Gao and Wang 2015). In the meantime, some experimental phenomena related to degradation behavior of critical current in Bi2223 superconducting tapes under torsional deformation have been observed (Shin and Katagiri 2003; Shin et al. 2003; Zou et al. 2015). Among them, it is found that the torsion strain does not significantly reduce the critical currents of Bi2223 tapes, when compared with cases of bending and/or tension loads (Shin and Katagiri 2003; Shin et al. 2003). On the other hand, the study on electromagnetic properties of superconducting cylinder has been reported (Jing et al. 2013). However, whether in theory or in experiment, the critical current in cylindrical superconducting body under the action of torque has never been addressed, let along in the elastic–plastic deformation stage.

In this paper, the model of torque dependence of critical current in a Bi2223/Ag composite is put forward and investigated. For simplicity, the considered Bi2223/Ag composite is assumed to be a cylindrical structure with circle cross section though the Bi2223 superconducting tape generally has a cross section more like an ellipse. Firstly, based on the hypothesis of planar section and elastic–plastic constitutive theory, the relation between torque and shear strain of the superconducting cylinder is derived. Secondly, using the linear weakening assumption, the relation between critical current density and shear strain is obtained in a clarity form. Finally, the torque dependence of critical current is numerical calculated and analyzed.

## The model of Bi2223/Ag composite concentric cylinder

**Fig. 1:**
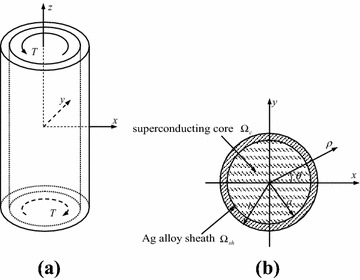
Schematic diagrams of superconducting model: **a** an infinitely long composite superconducting cylinder under torque, **b** cross section of the composite cylinder

As shown in Fig. 1, a Bi2223/Ag composite concentric cylinder consists of two components: the superconducting core and Ag alloy sheath. The radii of the total cross-section and superconducting core are $b = 1.05\;{\text{mm}}$ and $a = 1\;{\text{mm}}$, respectively. The core is composed of Bi2223 superconducting filament and the Ag matrix. For the considered composite concentric cylinder, the elastic moduli of these components are listed in Table 1 (Gou and Shen 2012).

**Table 1:** The volume fractions and elastic modulus of each component in the model

	Bi2223 (superconducting filament)	Ag (matrix in the core)	Ag alloy (sheath of model)
*V*	0.365	0.542	0.093
*G*(GPa)	21.9	28.2	32.1
	36.5		
	51.1		

Assume that the superconducting core consists of two components. Based on the simple rule of mixture, similar to the Young’s modulus in Ref. Gou and Shen (2012), the shearing modulus of the superconducting core *G*_*c*_ can be given as

1 $$G_{c} = G_{Bi} V^{\prime}_{Bi} + G_{Ag} V^{\prime}_{Ag} ,$$

where *G*_*Bi*_ and *G*_*Ag*_ are, respectively, the shearing moduli of Bi2223 filament and Ag matrix; *V*_*Bi*_^′^ and *V*_*Ag*_^′^ are the corresponding volume fractions of Bi2223 filament and Ag matrix in the superconducting core, which means that *V*_*Bi*_^′^ + *V*_*Ag*_^′^ = 1 holds true.

## Analysis of torque dependence of the critical current

### Relation between torque and maximum shear strain

As the composite concentric cylinder is subjected to a torque *T* at the ends of the cylinder body (see Fig. 1), the elastic deformation in the cylinder will take place. And with the increasing of *T*, the plastic deformation will further occur. Assume that in the total process of deformation, the Ag alloy sheath and superconducting core are connected ideally, and the hypothesis of planar section of the composite cylinder holds true. After that, the shear strain corresponding to radius coordinate *ρ* can be given as

2 $$\gamma \left( \rho \right) = \frac{{\gamma_{b} \rho }}{b},$$

where *γ*_*b*_ is the shear strain of the outmost layer (i.e., *ρ* = *b*).

Assuming that in the elastic–plastic deformation stages of both the superconducting core and the outer Ag alloy sheath, the linear strengthening elastic–plastic constitutive relations hold true, it is easily obtained that:

3 $$\left\{ {\begin{array}{*{20}l} {\tau_{sh}^{e} = {{G_{sh} \gamma_{b} \rho } \mathord{\left/ {\vphantom {{G_{sh} \gamma_{b} \rho } {b,}}} \right. \kern-0pt} {b,}}} \hfill &\quad {a \le \rho \le \rho^{\prime}_{sh} ,} \hfill \\ {\tau_{sh}^{p} = {{\alpha_{sh} G_{sh} \gamma_{b} \rho } \mathord{\left/ {\vphantom {{\alpha_{sh} G_{sh} \gamma_{b} \rho } b}} \right. \kern-0pt} b} + \left( {1 - \alpha_{sh} } \right)\tau_{shs} ,} \hfill & \quad{\rho^{\prime}_{sh} < \rho \le b,} \hfill \\ \end{array} } \right.$$

4 $$\left\{ {\begin{array}{*{20}l} {\tau_{c}^{e} = {{G_{c} \gamma_{b} \rho } \mathord{\left/ {\vphantom {{G_{c} \gamma_{b} \rho } {b,}}} \right. \kern-0pt} {b,}}} \hfill &\quad {0 \le \rho \le \rho^{\prime}_{c} ,} \hfill \\ {\tau_{c}^{p} = {{\alpha_{c} G_{c} \gamma_{b} \rho } \mathord{\left/ {\vphantom {{\alpha_{c} G_{c} \gamma_{b} \rho } b}} \right. \kern-0pt} b} + \left( {1 - \alpha_{c} } \right)\tau_{cs} ,} \hfill &\quad {\rho^{\prime}_{c} < \rho \le a,} \hfill \\ \end{array} } \right.$$

where

5 $$\left\{ {\begin{array}{*{20}l} {\rho^{\prime}_{sh} = {{\tau_{shs} b} \mathord{\left/ {\vphantom {{\tau_{shs} b} {\left( {G_{sh} \gamma_{b} } \right),}}} \right. \kern-0pt} {\left( {G_{sh} \gamma_{b} } \right),}}} \hfill \\ {\rho^{\prime}_{c} = {{\tau_{cs} b} \mathord{\left/ {\vphantom {{\tau_{cs} b} {\left( {G_{c} \gamma_{b} } \right).}}} \right. \kern-0pt} {\left( {G_{c} \gamma_{b} } \right).}}} \hfill \\ \end{array} } \right.$$

In Eqs. (3)–(5), *τ*_*sh*_^*e*^ and *τ*_*sh*_^*p*^ denote, respectively, the shear stresses of elastic phase and elastic–plastic phase of Ag alloy sheath, whilst *τ*_*c*_^*e*^ and *τ*_*c*_^*p*^ are the corresponding shear stresses of superconducting core. Also, *τ*_*shs*_ and *τ*_*cs*_ are, respectively, the shear yield stresses of the Ag alloy sheath and the superconducting core of the composite; *G*_*sh*_ and *G*_*c*_ are, respectively, the corresponding shearing moduli of the sheath and the superconducting core; *α*_*sh*_ and *α*_*c*_ are, respectively, the linear strengthening parameters of shearing moduli of the sheath and the superconducting core. It is pointed out that *α*_*sh*_ depends on material properties of the Ag sheath, whilst *α*_*c*_ is related to not only the material properties of Ag and Bi2223 but also the material components of them, and that in the following calculation process, both *α*_*sh*_ and *α*_*c*_ are assumed directly. In addition, $\rho^{\prime }_{sh}$ is a radial coordinate parameter introduced to distinguish the elastic phase zone and plastic phase zone of the sheath, and $\rho^{\prime }_{c}$ is the corresponding parameter introduced for the superconducting core.

Assume that as the shear stress at *ρ* = *a* in the superconducting core just reaches the corresponding yield stress *τ*_*cs*_, the shear strain of the outmost layer is *γ*_b_^*^. According to planar cross-section hypothesis, it is then obtained that

6 $$\gamma_{b}^{*} = \frac{{\tau_{cs} b}}{{G_{c} a}}.$$

For simplicity, it is further assumed that for the present composite cylinder, in the process of increasing torque, the superconducting core firstly steps into yield state, which, in fact, has been proved by numerical evaluations in the next section as well. Thus, as *γ*_*b*_ > *γ*_b_^*^, with the continuous increasing of *T*, on one hand, the superconducting core steps into the elastic–plastic state; on the other hand, the sheath will step into elastic–plastic state as well. Herein we introduce $\gamma_{b}^{\nabla } = \tau_{shs} /G_{sh}$ and $\gamma_{b}^{\Delta } = \tau_{shs} b/\left( {G_{sh}a} \right)$ to denote two critical states, which imply that $\tau_{sh} \left( b \right) = \tau_{shs}$ as $\gamma_{b} = \gamma_{b}^{\nabla }$, and $\tau_{sh} \left( a \right) = \tau_{shs}$ as $\gamma_{b} = \gamma_{b}^{\Delta }$.

Based on the mechanics of material, the following relation between the applied torque and shear strain of the outmost layer *γ*_*b*_ can be expressed as:

7 $$T = \left\{ {\begin{array}{*{20}l} {2\pi \int_{0}^{{\rho^{\prime}_{c} }} {\tau_{c}^{e} \rho^{2} d\rho } + 2\pi \int_{{\rho^{\prime}_{c} }}^{a} {\tau_{c}^{p} \rho^{2} d\rho } + 2\pi \int_{a}^{b} {\tau_{sh}^{e} \rho^{2} d\rho } ,} \hfill &\quad {\gamma_{b}^{*} \le \gamma_{b} < \gamma_{b}^{\nabla } ,} \hfill \\ {2\pi \int_{0}^{{\rho^{\prime}_{c} }} {\tau_{c}^{e} \rho^{2} d\rho } + 2\pi \int_{{\rho^{\prime}_{c} }}^{a} {\tau_{c}^{p} \rho^{2} d\rho } + 2\pi \int_{a}^{{\rho^{\prime}_{sh} }} {\tau_{sh}^{e} \rho^{2} d\rho } + 2\pi \int_{{\rho^{\prime}_{sh} }}^{b} {\tau_{sh}^{p} \rho^{2} d\rho } ,} \hfill &\quad {\gamma_{b}^{\nabla } \le \gamma_{b} < \gamma_{b}^{\Delta } ,} \hfill \\ {2\pi \int_{0}^{{\rho^{\prime}_{c} }} {\tau_{c}^{e} \rho^{2} d\rho } + 2\pi \int_{{\rho^{\prime}_{c} }}^{a} {\tau_{c}^{p} \rho^{2} d\rho } + 2\pi \int_{a}^{b} {\tau_{sh}^{p} \rho^{2} d\rho } ,} \hfill &\quad {\gamma_{b} \ge \gamma_{b}^{\Delta } .} \hfill \\ \end{array} } \right.$$

Substituting Eqs. (3) and (4) into Eq. (7), we can finally obtain the relation between torque and maximum shear strain as follows:

8 $$\frac{T}{2\pi } = \left\{ {\begin{array}{*{20}l} {\frac{{\left( {1 - \alpha_{c} } \right)\tau_{cs} a^{3} }}{3} + \frac{1}{4b}\left( {G_{c} \alpha_{c} a^{4} + G_{sh} b^{4} - G_{sh} a^{4} } \right)\gamma_{b} - \frac{{\left( {1 - \alpha_{c} } \right)\tau_{cs}^{4} b^{3} \gamma_{b}^{ - 3} }}{{12G_{c}^{3} }},} \hfill &\quad {\gamma_{b}^{*} \le \gamma_{b} < \gamma_{b}^{\nabla } ,} \hfill \\ \begin{aligned} \frac{1}{3}\left[ {\left( {1 - \alpha_{c} } \right)\tau_{cs} a^{3} + \left( {1 - \alpha_{sh} } \right)\tau_{shs} b^{3} } \right] + \frac{1}{4b}\left( {\alpha_{c} G_{c} a^{4} + \alpha_{sh} G_{sh} b^{4} - G_{sh} a^{4} } \right)\gamma_{b} \hfill \\ \quad - \frac{{b^{3} }}{12}\left[ {\left( {1 - \alpha_{c} } \right)G_{c}^{ - 3} \tau_{cs}^{4} + \left( {1 - \alpha_{sh} } \right)G_{sh}^{ - 3} \tau_{shs}^{4} } \right]\gamma_{b}^{ - 3} , \hfill \\ \end{aligned} \hfill &\quad {\gamma_{b}^{\nabla } \le \gamma_{b} < \gamma_{b}^{\Delta } ,} \hfill \\ \begin{aligned} \frac{1}{3}\left[ {\left( {1 - \alpha_{c} } \right)\tau_{cs} a^{3} + \left( {1 - \alpha_{sh} } \right)\tau_{shs} b^{3} - \left( {1 - \alpha_{sh} } \right)\tau_{shs} a^{3} } \right] \hfill \\ \quad + \frac{1}{4b}\left( {\alpha_{c} G_{c} a^{4} + \alpha_{sh} G_{sh} b^{4} - \alpha_{sh} G_{sh} a^{4} } \right)\gamma_{b} - \frac{{\left( {1 - \alpha_{c} } \right)\tau_{cs}^{4} b^{3} \gamma_{b}^{ - 3} }}{{12G_{c}^{3} }}, \hfill \\ \end{aligned} \hfill &\quad {\gamma_{b} \ge \gamma_{b}^{\Delta } .} \hfill \\ \end{array} } \right.$$

### Relation between critical current and maximum shear strain

When $\gamma_{b} \le \gamma_{b}^{*}$, the critical current density in the region of superconducting core, i.e., *Ω*_*c*_, is independent of *T*. However, with the further increasing of *T*, the plastic zone gradually occurs in *Ω*_*c*_ from *ρ* = *a*. The plastic zone will affect the size of critical current density. Inspired by both the elastic–plastic constitution model mentioned above and the fact that the effects of torsion strain on critical current in superconducting type are weaker than the ones of bending and/or tension loads, in the present study, an novel linear weakening model of the critical current density in the elastic–plastic state of Bi2223/Ag composite is set up as follows:

9 $$j\left( \rho \right) = \left\{ {\begin{array}{*{20}l} {j_{c} ,} \hfill &\quad {0 \le \rho \le \rho^{\prime}_{c} ,} \hfill \\ {j_{c} - \kappa \left( {\frac{{\gamma_{b} \rho }}{b} - \frac{{\tau_{cs} }}{{G_{c} }}} \right)j_{c} ,} \hfill &\quad {\rho^{\prime}_{c} < \rho \le \rho^{\prime\prime}_{c} ,} \hfill \\ 0 \hfill &\quad {\rho^{\prime\prime}_{c} < \rho \le a,} \hfill \\ \end{array} } \right.$$

where *j*_*c*_ denotes the critical current density with no plastic deformation. *κ* is an introduced weakening coefficient to be determined by relative experiments, which is directly assumed in the following calculation process as well. $$\rho^{\prime \prime }_{c} = \left( {G_{c} + \kappa \tau_{cs} } \right)b/\left( {\kappa G_{c} \gamma_{b} } \right)$$ satisfies $$j\left( {\rho^{\prime \prime }_{c} } \right) = 0$$. Equation (9) indicates that as $$\gamma_{b} \ge \gamma_{b}^{**} = \left( {G_{c} + \kappa \tau_{cs} } \right)b/\left( {\kappa G_{c} a} \right),\rho^{\prime \prime }_{c} \le a$$ holds true. It is remarked that the second equation of Eq. (9) describes the linear weakening model of critical current density in the inner plastic zone of superconducting core, the third equation implies the superconducting cylinder carrying no current in the outer plastic zone, and that the first equation corresponds to the ideal critical current density in the purely elastic zone. With the help of Eq. (9), the total critical current in the composite cylinder can be directly expressed as:

10 $$I_{c} = \left\{ {\begin{array}{*{20}l} {\pi a^{2} j_{c} ,} \hfill &\quad {0 \le \gamma_{b} < \gamma_{b}^{*} ,} \hfill \\ {\pi \rho^{\prime 2}_{c} j_{c} + 2\pi \int_{{\rho^{\prime}_{c} }}^{a} {\left[ {j_{c} - \kappa \left( {\frac{{\gamma_{b} \rho }}{b} - \frac{{\tau_{cs} }}{{G_{c} }}} \right)j_{c} } \right]} \rho d\rho ,} \hfill &\quad {\gamma_{b}^{*} \le \gamma_{b} < \gamma_{b}^{**} ,} \hfill \\ {\pi \rho^{\prime 2}_{c} j_{c} + 2\pi \int_{{\rho^{\prime}_{c} }}^{{\rho^{\prime\prime}_{c} }} {\left[ {j_{c} - \kappa \left( {\frac{{\gamma_{b} \rho }}{b} - \frac{{\tau_{cs} }}{{G_{c} }}} \right)j_{c} } \right]} \rho d\rho ,} \hfill &\quad {\gamma_{b} \ge \gamma_{b}^{**} .} \hfill \\ \end{array} } \right.$$

Define *I*_*c*0_ = *πa*^2^*j*_*c*_ as the critical current with no plastic zone occurring, we have

11 $$\frac{{I_{c} }}{{I_{c0} }} = \left\{ {\begin{array}{*{20}l} {1,} \hfill &\quad {0 \le \gamma_{b} < \gamma_{b}^{*} ,} \hfill \\ {\frac{{ - \kappa \tau_{cs}^{3} b^{2} }}{{3G_{c}^{3} \gamma_{b}^{2} a^{2} }} + \frac{{\kappa \tau_{cs} }}{{G_{c} }} - \frac{{2\kappa \gamma_{b} a}}{3b} + 1,} \hfill &\quad {\gamma_{b}^{*} \le \gamma_{b} < \gamma_{b}^{**} ,} \hfill \\ {\left( {\frac{{\tau_{cs} b}}{{G_{c} \gamma_{b} a}}} \right)^{2} + \frac{{\tau_{cs} b^{2} }}{{\kappa G_{c} \gamma_{b}^{2} a^{2} }} + \frac{{b^{2} }}{{3\kappa^{2} \gamma_{b}^{2} a^{2} }},} \hfill &\quad {\gamma_{b} \ge \gamma_{b}^{**} .} \hfill \\ \end{array} } \right.$$

Equation (11) reveals the relation between critical current of the composite cylinder and maximum shear strain *γ*_*b*_ of it.

Combining Eq. (11) with Eq. (8), we can finally obtain the torque dependence of the critical current of the present composite superconducting cylinder indirectly.

## Numerical results and discussion

**Fig. 2:**
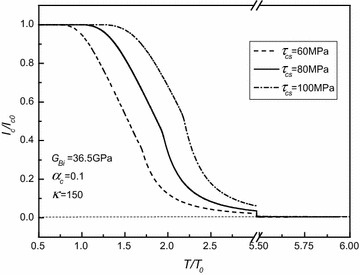
Normalized critical current versus normalized torque for different yield stresses of superconducting core as $G_{Bi} = 36.5\;{\text{GPa}}$, *α*_*c*_ = 0.1, and *κ* = 150

**Fig. 3:**
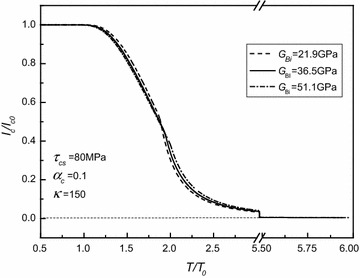
Normalized critical current versus normalized torque for different shear moduli of Bi2223 filament as $\tau_{cs} = 80\;{\text{MPa}}$, *α*_*c*_ = 0.1, and *κ* = 150

**Fig. 4:**
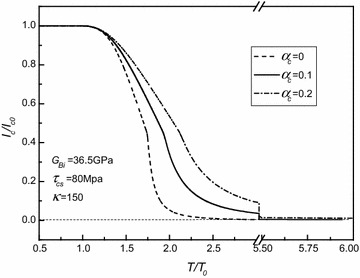
Normalized critical current versus normalized torque for different strengthening parameters of shearing elastic moduli of superconducting core as $G_{Bi} = 36.5\;{\text{GPa}}$, $\tau_{cs} = 80\;{\text{MPa}}$, and *κ* = 150

**Fig. 5:**
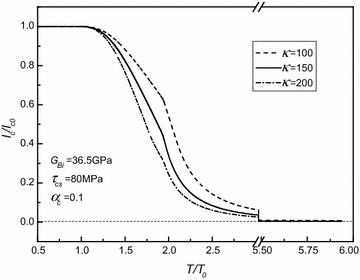
Normalized critical current versus normalized torque for different weakening coefficients of superconducting core related critical current density as $G_{Bi} = 36.5\;{\text{GPa}}$, $\tau_{cs} = 80\;{\text{MPa}}$, and *α*_*c*_ = 0.1

In order to deeply explore the effects of torque and material parameters on critical current, numerical results are plotted in Figs. 2, 3, 4 and 5, where $\tau_{shs} = 305\;{\text{MPa}}$ and *α*_*sh*_ = 0.2 are, respectively, assumed directly, and the applied torque is normalized by $T_{0} = \frac{{2\pi \left( {1 - \alpha_{c} } \right)\tau_{cs} a^{3} }}{3} + \frac{{\pi \tau_{cs} }}{{2G_{c} a}}\left( {G_{c} \alpha_{c} a^{4} + G_{sh} b^{4} - G_{sh} a^{4} } \right) - \frac{{\pi \left( {1 - \alpha_{c} } \right)\tau_{cs} a^{3} }}{6}$ with $\tau_{cs} \equiv 80\;{\text{MPa}}$, $G_{c} \equiv 31.53\;{\text{GPa}}$ calculated from $G_{Bi} = 36.5\;{\text{GPa}}$ and *α*_*c*_ ≡ 0.1 being adopted. In fact, *T*_0_ is the corresponding torque under which the inner superconducting core starts to yield.

As shown in the aforementioned figures, for given *τ*_*cs*_, *G*_*Bi*_, *α*_*c*_, and *κ*, there is an elastic limiting torque denoted as $T^{*} = \frac{{2\pi \left( {1 - \alpha_{c} } \right)\tau_{cs} a^{3} }}{3} + \frac{{\pi \tau_{cs} }}{{2G_{c} a}}\left( {G_{c} \alpha_{c} a^{4} + G_{sh} b^{4} - G_{sh} a^{4} } \right) - \frac{{\pi \left( {1 - \alpha_{c} } \right)\tau_{cs} a^{3} }}{6}$. As the applied torque *T* is smaller than T*, i.e., the superconducting core is in the stage of elastic deformation, the critical current is dependent of either the applied torque or the material property parameters *τ*_*cs*_, *G*_*Bi*_, *α*_*c*_, and *κ*. Then, for given *τ*_*cs*_, *G*_*Bi*_, *α*_*c*_, and *κ*, the critical current generally decreases with the increasing of torque (i.e., with the increasing of the plastic zone of the superconducting core). Finally, the critical current always tends to zero. Moreover, it is interesting to note that for each curve in these figures, there is an inflection point denoted as *T*^∇^. It has been demonstrated that *T*^∇^ corresponds to the torque after which the Ag alloy sheath starts to yield from the outmost layer. Moreover, by comparing all these figures, it is easily observed that both *τ*_*cs*_ and *α*_*c*_ have obvious effects on *T*^∇^, whilst the effects of *G*_*Bi*_ on *T*^∇^ are nearly insignificant, and *κ*, in fact, has no effect on the value of *T*^∇^. Figures 2, 3, 4 and 5 also indicate that T* depends on *τ*_*cs*_ more strongly than *G*_*Bi*_, and that T* is independent of either *α*_*c*_ or *κ*. In addition, it is seen that the varying trend of the present numerical results is similar to the one obtained for the Bi2223/Ag composite type by comparing Figs. 2, 3, 4 and 5 with Figs. 3, 4 in Ref. Shin et al. (2003). This, to a certain extent, means that our results are credible.

Also, Fig. 2 shows that, as expected, T* increases with the increasing of *τ*_*cs*_, and that for a given applied torque being larger than the maximum elastic limiting torque $T_{{\tau_{cs} ,\hbox{max} }}^{*}$, the critical current generally increases with the increasing of *τ*_*cs*_. This means that enlarging the superconducting material’s yielding limit stress can improve its critical current.

Figure 3 indicates that for a given applied torque being less than the minimum value of inflection points (i.e., $T_{{G_{Bi} ,\hbox{min} }}^{*}$) corresponding to three different shearing moduli of Bi2223 filament, the critical current slightly decreases with the increasing of *G*_*Bi*_. However, as the applied torque is larger than the maximum value of inflection points (i.e., $T_{{G_{Bi} ,\hbox{max} }}^{*}$) corresponding to the three different *G*_*Bi*_, the critical current firstly increases slightly with the increasing of it and finally tends to zero as well.

Figures 4 and 5 show that as the applied torque is larger than the corresponding elastic limiting torque, the critical current increases with the increasing of linear strengthening coefficient *α*_*c*_, whilst it decreases with the increasing of current weakening parameter *κ*.

## Brief conclusions

In this paper, a novel model of torque dependence of critical current in a Bi2223/Ag composite cylinder is established and analyzed, where a linear weakening relation between critical current density and shear strain is proposed. Numerical experiments reveal that the elastic limiting torque depends on the yielding limit of superconducting core more strongly than the shear modulus of Bi2223 filament. And it is independent of the introduced strengthening and/or weakening coefficients. On the other hand, during the stage of elastic–plastic deformation, enlarging either the superconducting material’s yielding limit stress or strengthening parameter of the superconducting core can improve the critical current of the composite cylinder, whilst adjusting the shear modulus of Bi2223 filament has nearly insignificant effect on the critical current of the considered composite superconducting body.
